# Related Factors of Anemia in Critically Ill Patients: A Prospective Multicenter Study

**DOI:** 10.3390/jcm11041031

**Published:** 2022-02-16

**Authors:** Raúl Juárez-Vela, Eva María Andrés-Esteban, Vicente Gea-Caballero, Juan Luis Sánchez-González, Pilar Marcos-Neira, Ainhoa Serrano-Lázaro, Gabriel Tirado-Anglés, Juan Carlos Ruiz-Rodríguez, Ángela Durante, Iván Santolalla-Arnedo, José Antonio García-Erce, Manuel Quintana-Díaz

**Affiliations:** 1 Doctoral Program in Medicine and Surgery, Autonomous University of Madrid, 28049 Madrid, Spain; raul.juarez@unirioja.es; 2 Research Institute Idi-Paz, PBM Group, 28046 Madrid, Spain; e.andres@live.com (E.M.A.-E.); vagea@universidadviu.com (V.G.-C.); mquintanadiaz@gmail.com (M.Q.-D.); 3 Department of Nursing, GRUPAC, University of La Rioja, 26004 Logroño, Spain; angela.durante@unirioja.es; 4 Department of Business Economics and Applied Economy, Faculty of Legal and Economic Sciences, University Rey Juan Carlos, 28032 Madrid, Spain; 5 Faculty of Health Sciences, International University of Madrid, 46010 Valencia, Spain; 6 Department of Nursing and Physiotherapy, University of Salamanca, 37007 Salamanca, Spain; juanluissanchez@usal.es; 7 Intensive Care Unit, Germans Trial I Pujol Hospital, 08916 Badalona, Spain; mpmarcosn@gmail.com; 8 Intensive Care Unit, University Clinic Hospital of Valencia, 46010 Valencia, Spain; asseranolazaro@gmail.com; 9 Intensive Care Unit, Royo Villanova Hospital, 50015 Zaragoza, Spain; gtiradoa@gmail.com; 10 Shock, Organ Dysfunction and Resuscitation Research Group, Intensive Care Department, Vall d’Hebron University Hospital, Vall d’Hebron, 08035 Barcelona, Spain; jcruiz@vhebron.net; 11 Blood and Tissue Bank of Navarra, Navarre Health Service, 31015 Pamplona, Spain; 12 Intensive Care Unit, La Paz University Hospital, 28046 Madrid, Spain

**Keywords:** anemia, blood, practice management, medical

## Abstract

Anemia is common in critically ill patients; almost 95% of patients admitted to intensive care units (ICUs) have hemoglobin levels below normal. Several causes may explain this phenomenon as well as the tendency to transfuse patients without adequate cause: due to a lack of adherence to protocols, lack of supervision, incomplete transfusion request forms, or a lack of knowledge about the indications, risks, and costs of transfusions. Daily sampling to monitor the coagulation parameters and the acid–base balance can aggravate anemia as the main iatrogenic factor in its production. We studied the association and importance of iatrogenic blood loss and other factors in the incidence of anemia in ICUs. We performed a prospective, observational, multicenter study in five Spanish hospitals. A total of 142 patients with a median age of 58 years (IQI: 48–69), 71.83% male and 28.17% female, were admitted to ICUs without a diagnosis of iatrogenic anemia. During their ICU stay, anemia appeared in 66.90% of the sample, 95 patients, (95% CI: 58.51–74.56%). Risk factors associated with the occurrence of iatrogenic anemia were arterial catheter insertion (72.63% vs. 46.81%, *p*-value = 0.003), venous catheter insertion (87.37% vs. 72.34%, *p*-value = 0.023), drainages (33.68% vs. 12. 77%, *p*-value = 0.038), and ICU stay, where the longer the stay, the higher the rate of iatrogenic anemia (*p*-value < 0.001). We concluded that there was a statistical significance in the production of iatrogenic anemia due to the daily sampling for laboratory monitoring and critical procedures in intensive care units. The implementation of patient blood management programs could address these issues.

## 1. Introduction

Anemia is very common in critically ill patients [1]; almost 95% of patients admitted to intensive care units (ICU) have hemoglobin levels below normal [2]. The CRIT study, a prospective, multicenter, observational cohort study in the United States that included 4892 ICU patients, reported that almost two-thirds of these patients had hemoglobin concentrations below 12 g/dL [3]. Although the etiology of anemia in critical patients is due to many factors which generate admission to the ICU, such as trauma, surgery, and gastrointestinal bleeding, it is also possible to determine anemia in critical patients who do not bleed. The pathogenesis of anemia in a non-bleeding, critical patient involves a combination of causes, the most important of which are sepsis; losses due to phlebotomy and minor procedures; decreased production of endogenous erythropoietin (EPO), and red blood cells with increased EPO resistance; destruction of red blood cells and functional iron deficiency associated with the immune system. In addition to the presence of neocytolysis, a hypothetical explanation for the selective lysis of young red blood cells (RBCs) (neocytes) associated with decreased plasma levels of erythropoietin (EPO), a phenomenon observed under experimental conditions that take place whenever a rapid RBC mass reduction is required [4]. In a critical patient, there could be a similar pathophysiological behavior within the first days of admission; there may be significant hemodilution due to the alteration of the hydric compartments after an initial intensive fluid therapy, where a plasma Hb has decreased but the total Hb mass has not been altered, with differences greater than 2 g per 100 mL being found between a measurement and the corrected one (carbon monoxide test) [5,6,7]. Since 2010, blood donations have fallen by 6.6% despite an increase in the global population, all while there has been an increase in the consumption of albumin and immunoglobulins of 58% and 99.6%, respectively, since 2012. This has led to shortages in blood, blood components, and blood products in some countries [8,9,10]. Nevertheless, it has been estimated that 5–58% of transfusions performed may be unnecessary, either due to clinician error or inaccurate transfusion volume [7]. Currently in Spain, after onco-hematology and emergency departments, intensive care services are one of the main consumers of blood components and blood products. The patient blood management (PBM) programs were developed to minimize unnecessary practices, reduce variabilities in clinical practice, decrease the rate of inappropriate and unnecessary transfusions, and promote the treatment of anemia as well as the correction of hemostasis, among others [11]. The inappropriate use of these limited resources is associated with increased respiratory distress, cardiac overload, iatrogenic infections, and hemolysis. This has led to longer hospital stays and complications derived from the transfusions themselves [12] as well as financial burdens and poor outcomes. In intensive care units (ICU), 40% of patients receive a transfusion of RBC concentrates during their stay at an average of 2–5 units [12,13]. Up to 30% of these patients with pre-transfusion Hb levels above 9 g/dL have been transfused without a clear indication [14], due to the characteristics of critical patients. The daily sampling of laboratories to control the coagulation parameters and the acid-base balance has resulted in weekly sampling between 340 and 660 mL of ICU patients, which can aggravate anemia as the main iatrogenic factor in its production [5,15]. In 1986, Burum referred to “vampire doctors” in his description of why iatrogenic anemia was the main factor that occurred in critical patients in the ICU [16,17] and how it could be improved with a PBM program. The objective of this study was to estimate the prevalence of iatrogenic anemia in critically ill patients and identify the factors associated.

## 2. Materials and Methods

### 2.1. Design and Population

This was an observational, prospective, multicenter study of all patients admitted to intensive care units (ICUs) during their stay at 5 Spanish hospitals.


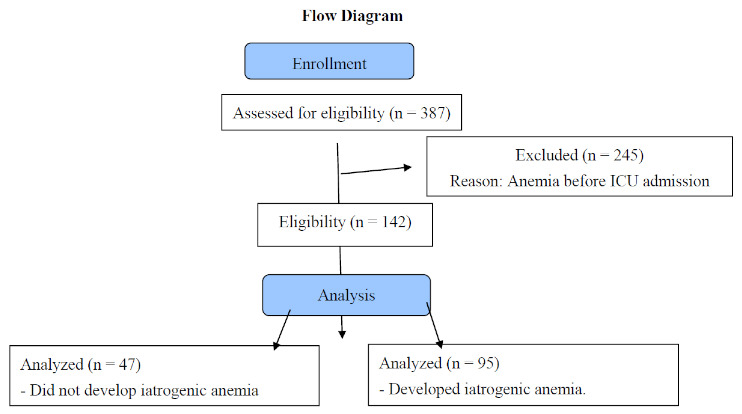


### 2.2. Variables and Clinical Criteria

According to World Heart Organization, anemia was defined as mild anemia: Hb < 13 g/dL in males, and Hb < 12 g/dL in females [18]. Regarding the indication for transfusion, anemia was classified according to the patient’s clinical condition: with hemodynamic repercussion: if the patient had hypotension and/or tachycardia; without hemodynamic repercussion: if the patient was stable. Renal function was assessed by serum creatinine (Cr) and urea levels, and acute renal failure was defined according to the AKIN criteria: increase in Cr in the ICU ≥ 0.3 mg/dL concerning baseline, or there was a percentage increase in Cr ≥ 50% [19].

In critical patients, we determined shock or cardiovascular failure when we had evidence of tissue hypoperfusion, which we detected following a decrease in venous oxygen saturation and/or an elevation of serum lactate beyond the presence, or not, of arterial hypotension MAP ≤ 65 mmHg and according to its enteropathogenic mechanism. The types of shock on admission were classified as septic, hypovolemic, hemorrhagic, or cardiogenic [20]. The prognostic scales used to predict ICU mortality were the sequential organ failure assessment (SOFA), which also assesses organ failure on ICU admission, and acute physiology and chronic health evaluation (APACHE II), which also estimates severity in the first 24 h of ICU admission [21]. SOFA predicts 20% mortality with an admission score of 6 points, 50% with 12 points and 90% when >17. APACHE II determines that a score of 25 predicts 50% mortality, and above 35, 80% mortality.

Demographic and clinical variables such as age, sex, and previous comorbidities were also collected. In addition, risk factors for anemia were collected, such as catheter insertion, drainage, total daily blood volume drawn in the critically ill patient, ICU stay, and exitus.

### 2.3. Statistical Procedures

Convenience sampling was carried out in ICUs located in Madrid (La Paz), Aragon (Rollo Villanova), Catalonia (Germans Trial I Pujol and Vall d’Hebron), and Valencia (Clínico). All patients who met the following inclusion criteria were selected for the study: those over 16 years old, having been admitted to the ICU for any reason, and with a stay of more than one day (i.e., 24 h). We did not exclude any participants. 

For the description of the main variables, frequency distribution was used in the case of qualitative variables, while the median and interquartile range was used for quantitative variables, due to the non-normality of the variables. The normality test used was the Shapiro–Wilks test. To compare previous demographic and clinical variables, as well as the treatments and other risk factors between patients who had anemia and those who did not, the chi-squared test was used when the variable was qualitative and the non-parametric Mann–Whitney U test for quantitative variables. Multivariate analysis was performed using binary logistic regression with the forward conditional method. The dependent variable was the presence of anemia, and the independent variables were all the variables that were statistically significant in the bivariate analysis or the clinical implication of which could be plausible. Finally, the independent variables were the following: SOFA, APACHE II, shock, blood volume collected (mL), arterial catheters inserted, venous catheters inserted, drainage, and ICU stay. 

**Figure 1:**
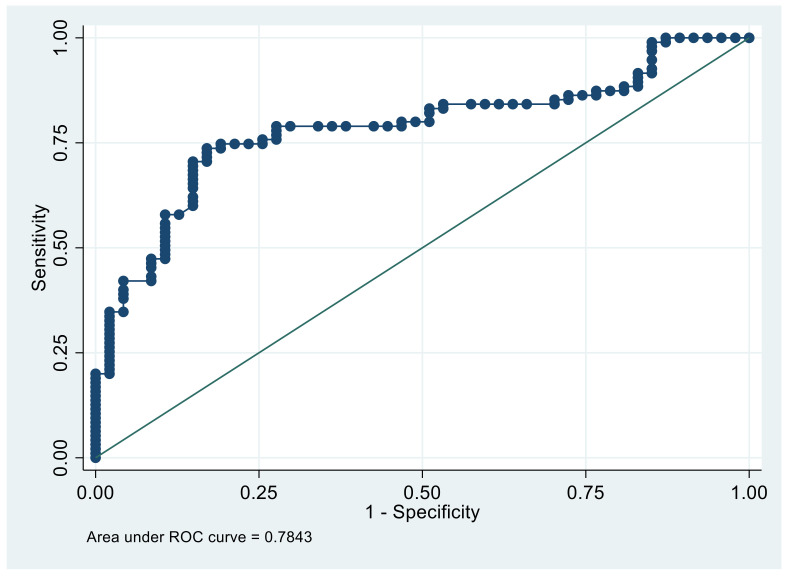
Roc Curve. Validation of multivariate model. (Hosmer–Lemeshow test).

In order to validate the multivariate model, calibration was performed using the Hosmer–Lemeshow statistical test. The discriminatory power was assessed using the area under the receiver-operating characteristic (ROC) curve (see Figure 1) obtained by analyzing the probability of the value predicted by the multivariate model. The results of the multivariable model were presented by odds ratio (OR) and its 95% confidence interval (CI 95%).

## 3. Results

Data were collected from a total of 387 patients, of which the prevalence of anemia upon ICU admission was 63.31% (95%, CI: 58.29–68.12%). Only 142 patients were admitted to our study without a diagnosis of anemia with a median age of 58 years (IQI: 48–69), 71.83% male and 28.17% female.

The predominant reason for admission to the ICU was surgery (85.92%). Medical reasons accounted for only 5.63% of ICU admissions (Table 1). Of the patients who did present shock upon admission, the most frequent causes were septic shock (9.86%) and cardiogenic shock (8.45%). There were significant differences in the distribution of the different types of shock between patients who progressed to anemia in the ICU and those who did not. In this regard, we observed that the rate of patients without shock was lower in patients with anemia during their evolution (65.26% vs. 89.36%).

We also found significant differences between the SOFA and APACHE II severity scales in patients with and without progression to anemia. In both cases, patients with progression to anemia had the highest scores on both scales, indicating greater severity, as shown in Table 1. There were no significant differences found in either the sex or the age of the patients. Nor were there significant differences in comorbidities prior to admission to the ICU.

Concerning extractions performed in the ICU, the percentage of patients admitted to the ICU who had more than three tubes extracted daily was very high. Specifically, 87.68% in patients with SOFA 0–3 pts; 91.89% in patients with SOFA 4–7 pts; 93.5% in patients with SOFA 8–11 pts; and 95.83% in patients with SOFA > 11 pts. 

The volume of blood collected on the day of data collection had a median of 28.45 mL (IQI: 18.20–55.35), with significant differences between patients who progressed to anemia and those who did not, *p*-value = 0.031. 

Specifically, patients without anemia had a blood collection of 0–30 mL in 68.09% versus 43.16% of patients with anemia (Table 2). Most notably, a collection above 60 mL was performed in 6.38% of patients without anemia versus 32.63% of patients with anemia. Therefore, 28/95 patients with anemia required blood transfusion, and 13 of those required more than 4 transfusions.

The risk factors associated with the occurrence of iatrogenic anemia were arterial catheter insertion (72.63% vs. 46.81%, *p*-value = 0.003), venous catheter insertion (87.37% vs. 72.34%, *p*-value = 0.023), drainage (33.68% vs. 12. 77%, *p*-value = 0.038), and ICU stay, where the longer the stay, the higher the rate of iatrogenic anemia (*p*-value < 0.001), as shown in Table 2. There were no significant differences in patient mortality (*p*-value = 0.206).

The multivariate analysis of the factors associated with the appearance of iatrogenic anemia during patients’ stay in the ICU included the admission scale at 24 h of admission (OR = 1.14, 95% CI: 1.05–1.24), the volume of blood collected daily due to extractions carried out via ICU services (OR = 1.99, 95% CI: 1.03–3.82), and the length of stay in the ICU, especially after half a month, where a longer stay was associated with the appearance of anemia, as shown in Table 3.

The Hosmer–Lemeshow test was performed in order to test the goodness of the regression model in accordance with the results performed with c-statistic, both indicators, and with a *p*-value of 0.294 for the Hosmer–Lemeshow test and 0.784 for the c-statistic (Figure 1), indicating that the proposed model was a good predictive model.

## 4. Discussion 

The aim of this study was to estimate the association and importance of anemia and iatrogenic blood loss with related factors. Our results indicated that in a cohort of 142 subjects admitted to the ICU, 66.90% of them had anemia during their stay. Likewise, the rate of anemia increased the longer they remained in the ICU. In a recent study published by Warner et al. [22], in a cohort of 6901 adults hospitalized in the ICU, 41% had anemia before hospitalization, a value slightly lower than that obtained in our study. However, as we have commented previously, the longer they remained in the ICU, the more the prevalence of anemia increased.

One of the predominant medical indications in patients hospitalized in ICUs is the treatment and recovery of anemia. There is a paucity of data on recovery from anemia in patients hospitalized in ICUs. There have been studies that affirmed that more than 50% of survivors with anemia on discharge from the ICU had persistent anemia at 6 months [23]. One of the possible explanations for this phenomenon may be an inflammatory process mediated by C-reactive protein and interleukin-6 that could alter erythropoiesis [24].

Regarding the risk factors associated with the appearance of anemia, these included the presence of arterial and venous catheters, drains, the length of stay in the ICU, and, especially, the amount of blood volume extracted from the critical patient. Our results showed that the volume of the blood extracted daily (OR = 1.99, 95% CI: 1.03–3.82) from critical patients could be one of the main risk factors associated with the appearance of anemia. In addition, the risk was higher in patients admitted to the ICU as they are more closely observed and it is necessary to obtain blood samples for subsequent analysis, which may increase their vulnerability to anemia. In our multivariate analysis, there was a clear relationship (*p*-value 0.038) between the volume of blood collected and the production of iatrogenic anemia. Considering the systematic review carried out by Whitehead et al. [25], our results agreed, and we likewise emphasized the use of blood conservation systems to eliminate blood waste when drawing blood for analysis. Our results are in correlation with those established by Salisbury et al. [26], where an 18% risk increase for anemia was correlated to every 50 mL of blood drawn. While a healthy person can tolerate a loss of approximately 500 mL of blood in a donation, critical patients develop anemia at much lower loss volumes. The relationship (*p*-value 0.031) indicates the importance of the limit of collecting blood in the iatrogenic blood loss in daily procedures. The use of pediatric devices for collected blood is common in ICUs in order to minimize the impact of iatrogenic blood loss in critically ill patients. A total of 32.63% of our patients developed anemia with the extraction of more than 60 mL. Currently, modern blood analyzers require around 100–200 microliters of blood for analysis while blood draws are performed with standard volume tubes ranging from 4 to 6 mL in volume. Several studies [5,15] have shown that weekly blood loss due to laboratory blood samples for coagulation control, the acid–base balance for the detection of iatrogenic infections, or for the monitoring of organ function varies from 340 to 660 mL in ICU patients. The appearance of anemia in these types of patients coincides with a series of deficiencies, such as decreased mobility [27,28], muscle weakness [29], and global cognitive impairment [30]. Therefore, appropriate treatment is essential to avoid these deficiencies. 

When using standard volume tubes, more than 90% of the blood is discarded; therefore, the volume of blood collected could be reduced without reducing the number of diagnostic tests, which could improve outcomes for critical patients [31].

Our findings suggested the association and importance of iatrogenic blood loss and different factors in critically ill patients. There was a statistical significance in the onset of iatrogenic anemia due to the daily blood draws for laboratory monitoring, which could be improved with the implementation of PBM programs as well, as has been demonstrated in different studies [32,33,34] 

Our study had some limitations. Firstly, this study had a prospective design with a small number of included patients and a short follow-up duration. However, this is the first study to evaluate iatrogenic anemia in critical patients in five intensive care units in Spain and the first to examine the need for PBM programs to reduce iatrogenic procedures.

Secondly, we have to consider the relationship between inflammation and anemia. Finally, this study does not delve into the cause–effect relationship due to its epidemiological design. We must bear in mind that some statistically significant relationships may have different risk factors.

## 5. Conclusions

We concluded that anemia and RBC transfusions in critically ill patients were due, at least in part, to unnecessary diagnostic blood sampling. Efforts to optimize both the frequency and the volume of tests will avoid wasting large amounts of blood and improve clinical outcomes for patients in ICUs around the world. In addition, setting up PBM programs as WHO has recommended for the minimization of bleeding and iatrogenic blood loss will become the gold standard. Further expanded studies are necessary to determine the real impact of clinical iatrogenic blood loss and its impact on critical patients.

## Figures and Tables

**Table 1:** Demographics and clinical variables in ICU admission.

		All Patients (*n* = 142)	Not Developed Iatrogenic Anemia (*n* = 47)	Developed Iatrogenic Anemia (*n* = 95)	*p*-Value
Sex					0.274
	Males	102 (71.83%)	31 (65.96%)	71 (74.74%)	
	Females	40 (28.17%)	16 (34.04%)	24 (25.26%)	
Age					0.986
	0–44	28 (19.72%)	9 (19.15%)	19 (20.00%)	
	45–54	33 (23.24%)	10 (21.28%)	23 (24.21%)	
	55–64	27 (19.01%)	10 (21.28%)	17 (17.89%)	
	65–74	32 (22.54%)	11 (23.40%)	21 (22.11%)	
	>75	22 (15.49%)	7 (14.89%)	15 (15.79%)	
Comorbidities					
	Previous treatment with NSAIDs	14 (9.86%)	6 (12.77%)	8 (8.42%)	0.414
	chronic anemia	2 (1.41%)	0 (0.00%)	2 (2.11%)	0.316
	haemato-oncological disease	9 (6.34%)	2 (4.26%)	7 (7.37%)	0.474
	heart failure	13 (9.15%)	7 (14.89%)	6 (6.32%)	0.095
	ischemic heart disease	16 (11.27%)	7 (14.89%)	9 (9.47%)	0.336
	hepatopathy	1 (0.70%)	0 (0.00%)	1 (1.05%)	0.480
	chronic occlusive pulmonary disease	17 (11.97%)	8 (17.02%)	9 (9.47%)	0.192
	chronic renal insufficiency	10 (7.04%)	1 (2.13%)	9 (9.47%)	0.107
Shock					0.008
	No shock	104 (73.24%)	42 (89.36%)	62 (65.26%)	
	Septic shock	14 (9.86%)	0 (0.00%)	14 (14.74%)	
	Hemorrhagic shock	4 (2.82%)	0 (0.00%)	4 (4.21%)	
	Hypovolemic shock	5 (3.52%)	0 (0.00%)	5 (5.26%)	
	Cardiogenic shock	12 (8.45%)	3 (6.38%)	9 (9.47%)	
	Others	3 (2.11%)	2 (4.26%)	1 (1.05%)	
SOFA in ICU admission (median, QI)		5 (2–7)	4 (2–6)	6 (3–8)	0.033
APACHE (into 24 h)		16 (11–21)	13 (9–18)	16 (11–23)	0.009
Diagnosis					0.137
	Cardiovascular pathology	14 (9.86%)	9 (19.15%)	5 (5.26%)	
	Respiratory pathology	6 (4.23%)	1 (2.13%)	5 (5.26%)	
	Neurological pathology	66 (46.48%)	22 (46.81%)	44 (46.32%)	
	Infectious pathology	11 (7.75%)	3 (6.38%)	8 (8.42%)	
	Surgery	19 (13.38%)	4 (8.51%)	15 (15.79%)	
	Other	26 (18.31%)	8 (17.02%)	18 (18.95%)	
Admission					0.132
	Medical	8 (5.63%)	2 (4.26%)	6 (6.32%)	
	Surgery	122 (85.92%)	44 (93.62%)	78 (82.11%)	
	Other	12 (8.45%)	1 (2.13%)	11 (11.58%)	

ICU = Intensive Care Unit. NSAIDs = Non-steroidal anti-inflammatory drugs. SOFA = Sequential Organ Failure Assessment Score QI = Quartile Interval. APACHE = Acute Physiology and Chronic Health Evaluation.

**Table 2:** Treatments, risk factors, and ICU exitus related to iatrogenic anemia.

			All Patients (*n* = 142)	Not Developed Iatrogenic Anemia (*n* = 47)	Developed Iatrogenic Anemia (*n* = 95)	*p*-Value
Treatments						
	Blood volume collected (mL)					0.031
		0–30 mL	73 (51.41%)	32 (68.09%)	41 (43.16%)	
		31–60 mL	35 (24.65%)	12 (25.53%)	23 (24.21%)	
		>60 mL	34 (23.94%)	3 (6.38%)	31 (32.63%)	
	Transfusion of red blood cell concentrates (%)		28 (19.72%)	0 (0.00%)	28 (29.47%)	<0.001
	The number of red blood cells concentrates					<0.001
		None	113 (79.48%)	47 (100.00%)	66 (69.47%)	
		1 or 2	10 (7.04%)	0 (0.00%)	10 (10.53%)	
		3 or 4	6 (4.23%)	0 (0.00%)	6 (6.32%)	
		>4	13 (9.15%)	0 (0.00%)	13 (13.68%)	
	Fe administration (%)		6 (4.23%)	1 (2.13%)	5 (5.26%)	0.386
Risk factors						
	Arterial catheters inserted (%)		91 (64.08%)	22 (46.81%)	69 (72.63%)	0.003
	Venous catheters inserted (%)		117 (82.39%)	34 (72.34%)	83 (87.37%)	0.023
	Drainage (%)		38 (26.76%)	6 (12.77%)	32 (33.68%)	0.038
	Presence of hemofilters (%)		7 (4.93%)	0 (0.00%)	7 (7.37%)	0.056
	ICU stay					<0.001
		0–6 days	30 (21.13%)	17 (36.17%)	13 (13.68%)	
		7–16 days	42 (29.58%)	20 (42.55%)	22 (23.16%)	
		17–31 days	34 (23.94%)	8 (17.02%)	26 (27.37%)	
		More than 31 days	36 (25.35%)	2 (4.26%)	34 (35.79%)	
Outcome						
	Exitus (%)		12 (8.45%)	2 (4.26%)	10 (10.53%)	0.206

**Table 3:** Multivariate analysis.

		OR	CI 95%	*p*-Value
APACHE (into 24 h)		1.14	1.05–1.24	0.002
Blood volume collected (mL)		1.99	1.03–3.82	0.038
ICU stay				
	0–6 days	--	--	--
	7–16 days	1.27	0.44–3.62	0.650
	17–31 days	3.65	1.12–11.85	0.031
	More than 31 days	15.28	2.26–103.01	0.005

OR = Odds Ratio. CI = Confidence Interval.

## Data Availability

The datasets analyzed during the current study are available from the corresponding author upon reasonable request.

## References

[1] Von Ahsen N., Muller C., Serke S., Frei U., Eckardt K.U. (1999). Important role of nondiagnostic blood loss and blunted erythropoietic response in the anemia of medical intensive care patient. Crit. Care Med..

[2] Hajjar A., Auler J.O., Santos L., Galas F. (2007). Blood tranfusion in critically ill patients: State of the art. Clinics.

[3] Corwin H.L., Gettinger A., Pearl R.G., Fink M.P., Levy M.M., Abraham E., MacIntyre N.R., Shabot M.M., Duh M.S., Shapiro M.J. (2004). The CRIT Study: Anemia and blood transfusion in the critically ill—Current clinical practice in the United States. Crit. Care Med..

[4] Risso A., Ciana A., Achilli C., Antonutto G., Minetti G. (2014). Neocytolysis: None, one or many? A reappraisal and future perspectives. Front. Physiol..

[5] Nguyen B.V., Bota D.P., Melot C., Vincent J.L. (2003). Time course of hemoglobin concentrations in nonbleeding intensive care unit patients. Crit. Care Med..

[6] Vincent J.L., Baron J.F., Reinhart K., Gattinoni L., Thijs L., Webb A., Meier-Hellmann A., Nollet G., Peres-Bota D., ABC Investigators (2002). Anemia and blood transfusion in critically ill patients. JAMA.

[7] Quintana-Díaz M. (2017). Appropriate use of red blood cell transfusion in emergency departments: A study in five emergency departments. Blood Transfus..

[8] World Health Organization (2008). Blood Transfusion Safety. Global Database on Blood Safety.

[9] García-Erce J.A., Jericó C., Abad-Motos A., Rodríguez García J., Antelo Caamaño M.L., Domingo Morera J.M., Sola Lapeña C., Arroyo J.L., Fernández Fuertes F., Zalba Marcos S. (2021). Quintana Díaz MPBM: Now more than ever necessary. Rev. Española Anestesiol. Reanim..

[10] Jericó C., García-Erce J.A. (2021). Anemia and transfusion, “with or without you”. Med. Clin..

[11] World Health Organization (2020). Action Framework to Advance Universal Access to Safe, Effective and Quality Assured Blood Products. Organización Mundial de la Salud. https://www.who.int/publications/i/item/action-framework-to-advance-uas-bloodprods-978-92-4-000038-4.

[12] Long B., Koyfman A. (2016). Red Blood Cell Transfusion in the Emergency Department. J. Emerg. Med..

[13] Noval S.L., Gómez M.M., García A.C. (2004). Transfusion en el paciente crítico. Med. Intensiva.

[14] Leal-Noval S.R., Arellano-Orden V., Maestre-Romero A., Muñoz-Gómez M., Fernández-Cisneros V., Ferrándiz-Millón C., Corcia Y. (2011). Impact of national transfusion indicators on appropriate blood usage in critically ill patients. Transfusion.

[15] Silver M.J., Li Y.H., Gragg L.A., Jubran F., Stoller J.K. (1993). Reduction of blood loss from diagnostic sampling in critically ill patients using a blood-conserving arterial line system. Chest.

[16] Burnum J.F. (1986). Medical vampires. N. Engl. J. Med..

[17] Ripollés-Melchor J., Jericó-Alba C., Quintana-Díaz M., García-Erce J.A. (2018). Del ahorro de sangre al patient blood management. Med. Clin..

[18] World Health Organization Hemoglobin Concentrations to Diagnose Anemia and Assess Its Severity. 2011. World Health Organization. https://apps.who.int/iris/handle/10665/85842.

[19] Mehta R.L., Kellum J.A., Shah S.V., Molitoris B.A., Ronco C., Warnock D.G., Levin A. (2007). Acute Kidney Injury Network: Report of an initiative to improve outcomes in acute kidney injury. Crit. Care.

[20] Minne L., Irwin R., Rippe J. (2006). Intensive Care Medicine.

[21] Abu-Hanna A., de Jonge E. (2008). Evaluation of SOFA-based models for predicting mortality in the ICU: A systematic review. Crit. Care.

[22] Warner M.A., Hanson A.C., Frank R.D., Schulte P.J., Go R.S., Storlie C.B., Kor D.J. (2020). Prevalence of and Recovery from Anemia Following Hospitalization for Critical Illness among Adults. JAMA Netw. Open.

[23] Bateman A.P., McArdle F., Walsh T.S. (2009). Time course of anemia during six months follow up following intensive care discharge and factors associated with impaired recovery of erythropoiesis. Crit. Care Med..

[24] Griffith D.M., Vale M.E., Campbell C., Lewis S., Walsh T.S. (2016). Persistent inflammation and recovery after intensive care: A systematic review. J. Crit. Care.

[25] Whitehead N.S., Williams L.O., Meleth S., Kennedy S.M., Ubaka-Blackmoore N., Geaghan S.M., Nichols J.H., Carroll P., McEvoy M.T., Gayken J. (2019). Interventions to prevent iatrogenic anemia: A Laboratory Medicine Best Practices systematic review. Crit. Care.

[26] Salisbury C., Johnson L., Purdy S., Valderas J.M., Montgomery A.A. (2011). Epidemiology and impact of multimorbidity in primary care: A retrospective cohort study. Br. J. Gen. Pract..

[27] Penninx B.W., Pahor M., Cesari M., Corsi A.M., Woodman R.C., Bandinelli S., Guralnik J.M., Ferrucci L. (2004). Anemia is associated with disability and decreased physical performance and muscle strength in the elderly. J. Am. Geriatr. Soc..

[28] Penninx B.W., Guralnik J.M., Onder G., Ferrucci L., Wallace R.B., Pahor M. (2003). Anemia and decline in physical performance among older persons. Am. J. Med..

[29] Thein M., Ershler W.B., Artz A.S., Tecson J., Robinson B.E., Rothstein G., Liede A., Gylys-Colwell I., Lu Z.J., Robbins S. (2009). Diminished quality of life and physical function in community-dwelling elderly with anemia. Medicine.

[30] Andro M., Le Squere P., Estivin S., Gentric A. (2013). Anaemia and cognitive performances in the elderly: A systematic review. Eur. J. Neurol..

[31] Siegal D.M., Manning N., Jackson Chornenki N.L., Hillis C.M., Heddle N.M. (2020). Devices to reduce the volume of blood taken for laboratory testing in ICU patients: A systematic review. J. Intensive Care Med..

[32] García-Erce J.A., Romón-Alonso Í., Jericó C., Domingo-Morera J.M., Arroyo-Rodríguez J.L., Sola-Lapeña C., Bueno-Cabrera J.L., Juárez-Vela R., Zalba-Marcos S., Abad-Motos A. (2021). Blood donations and transfusions during the COVID-19 pandemic in spain: Impact according to autonomous communities and hospitals. Int. J. Environ. Res. Public Health.

[33] Quintana-Díaz M., Andrés-Esteban E.M., Sánchez-Serrano J., Martínez-Virto A., Juárez-Vela R., García-Erce J.A. (2020). Transfusions in the Emergency department: More than a blood transfusion. Rev. Clin. Esp..

[34] Quintana-Diaz M., Nanwani-Nanwani K., Marcos-Neira P., Serrano-Lázaro A., Juarez-Vela R., Andrés-Esteban E.M. (2020). Epidemiología de la transfusión sanguínea en los Servicios de Medicina Intensiva en España: «Transfusion Day». Med. Intensiva.

